# Breaking barriers: harnessing artificial intelligence for a stigma-free, efficient HIV prevention assessment among adults in South Africa

**DOI:** 10.3389/fdgth.2025.1731002

**Published:** 2026-02-02

**Authors:** Caroline Govathson, Candice Chetty-Makkan, Ross Greener, Sasha Frade, Dino Rech, Sarah Morris, Yohann Richard, Rouella Mendonca, Natalie Maricich, Lawrence Long, Sophie Pascoe

**Affiliations:** 1 Health Economics and Epidemiology Research Office, Faculty of Health Sciences, University of the Witwatersrand, Johannesburg, South Africa; 2 Audere Africa, Johannesburg, South Africa; 3 Audere, Seattle, WA, United States; 4 Department of Global Health, Boston University School of Public Health, Boston, MA, United States

**Keywords:** artificial intelligence, HIV, large language model, prevention, South Africa

## Abstract

**Background:**

The quality of interactions between healthcare providers (HCPs) and recipients of care (ROC) are important for assessing HIV vulnerability and determining PrEP eligibility. However, these conversations are often limited because of high HCP workload and time constraints. Conversational agents powered by large language models (LLM) offer a promising solution to support such interactions. We explored the potential of an LLM-powered app prototype “Your Choice” to engage people who have recently tested HIV negative in discussions on HIV prevention and PrEP.

**Objective:**

We assessed usability, acceptability, feasibility and appropriateness of the LLM-powered app prototype “Your Choice” in identifying HIV vulnerability and summarizing relevant information to support HCPs in designing personalised HIV prevention plans.

**Methods:**

Using a human-centred design (HCD) approach, we co-developed the “Your Choice” app. Between August 2023 and March 2024, we conducted surveys with ROCs and HCPs following app use. We also analysed app-user interactions for themes and content patterns. Quantitative data were analysed descriptively; qualitative data underwent content analysis.

**Results:**

We enrolled a total of 150 participants (130 ROC + 20 HCPs). Among the ROCs, most were male (77/130; 59.2%) and aged 18–34 years (93/130; 71.5%). ROCs rated the app highly acceptable and appropriate (AIM and IAM scores >4.9/5), with excellent usability scale scores (SUS >92/100, indicating exceptional usability). HCPs (*n* = 16) experienced in PrEP service delivery reviewed the summaries and the interactions with ROC from the app. In the pre-evaluation survey, only one expressed concern about trusting technology (1/16; 6.3%) and two thirds preferred their own judgement for ROC care (11/16; 68.8%). HCPs rated the app with a high usability scale score (SUS 78/100, indicating good usability). The app fostered open, stigma-free discussions on sex, sexuality, and HIV prevention.

**Conclusion:**

An LLM-powered conversational agent like “Your Choice” shows promise for private, stigma-free HIV prevention support, including PrEP uptake, supporting the decision-making around PrEP initiation. It can also help providers deliver more targeted care. Future research should address technological trust and clinical integration.

## Introduction

To optimise diagnosis, treatment, and ongoing care, healthcare providers (HCPs) and recipients of care (ROCs) need to exchange appropriate and accurate information (1, 2). Health-related information is often sensitive, and sharing necessitates empathy, compassion, and mutual trust between HCP and ROC (2–4). Yet, fostering these conversations can be difficult for multiple reasons, including high patient loads, limited time, and complex, sensitive subject matter (5, 6). In South Africa, all three reasons are relevant, given HCP shortages in the public sector and the high HIV prevalence in the population (3, 4, 7). Without supportive HCP-ROC interactions, ROC may feel afraid, ashamed, or unwilling to communicate necessary information about their health behaviours, needs, concerns, and preferences (8–11). Strategies for bridging this communication gap between ROCs and HCPs are critical to the success of patient-centred care.

Artificial intelligence (AI) has the potential to help bridge this gap (8, 9, 11). One approach is leveraging generative AI tools, specifically Large Language Models (LLMs), which have demonstrated a remarkable ability to answer questions, summarise, and provide information (12–15). Given their demonstrated capacity for empathy (16, 17) in recent research, LLMs could serve as conversational agents to enhance communication between HCP and ROC through personalised, empathetic conversations.

Research on attitudes and preferences regarding AI and its capabilities for empathetic communication has shown varied results (18, 19). While some studies show HCPs are preferred over AI conversational agents (20, 21), another study suggests AI conversational agents could offer more empathetic responses than human providers (22). Although the potential benefits of AI use in healthcare are promising, more evidence supporting its potential impact in resource-constrained settings is needed (23–25). To date, most studies have predominantly focused on higher resource settings, addressing conditions prevalent in these environments (26–29). Concerns have been raised about the ability of AI conversational agents to fully capture the nuances of healthcare systems, access barriers, and cultural norms in settings like South Africa (30) and the potential impact of these limitations on patient care requires careful examination. Before questions on scalability or health outcomes can be addressed, we must demonstrate proof of concept with a prototype showing that it works in principle, is safe, appropriate, and acceptable. In this research, we assessed the usability, acceptability, feasibility, and appropriateness of an LLM conversational agent for HIV vulnerability screening, information provision, and counselling.

South Africa, with its large HIV programme, constrained human resources, and high smartphone penetration, represents a context where AI counselling tools could both be impactful and feasibly implemented (31, 32).

While South Africa's HIV treatment program has substantially increased life expectancy, the epidemic persists (31); achieving control requires expanding access to effective HIV prevention (33, 34). ROCs that test HIV negative are candidates for various HIV prevention modalities. One of the most effective being oral daily PrEP, which is offered free at the point of care within the South African primary health care system (35). Increased PrEP uptake among vulnerable populations is important to meet HIV infection reduction targets (33, 34, 36). Productive discussions between HCP and ROC about HIV prevention are plagued by many barriers in the South African context. These barriers include the limited number of HCPs, stigma associated with sexual behaviour, and lack of knowledge, amongst others (37–39).

This study evaluates a generative AI intervention for HIV prevention in a low- and middle-income country. We co-designed and tested a prototype LLM-powered conversational agent, “Your Choice”, with ROCs and HCPs in a non-clinical setting in South Africa. The conversational agent was designed to facilitate conversations around HIV prevention, in particular to determine potential benefit from and readiness for HIV pre-exposure prophylaxis (PrEP) after a negative HIV test, and provide the HCP with summarised information to boost the performance of their in-person interaction with the ROC.

## Methods

Between August and October 2023, we enrolled the initial 100 participants in our ROC study to test our initial text-only LLM-powered counselling prototype in a non-clinical setting. The final 30 ROC participants were enrolled between January and March 2024 to evaluate the second LLM-powered app prototype that included text-to-audio and audio-to-text features. HCP participants were enrolled between 20 September and 20 November 2023 and reviewed output based on the first prototype only. The LLM-powered app was assessed for usability, acceptability, and appropriateness among ROCs. In addition to these assessments, HCPs evaluated its feasibility.

### Intervention: LLM-powered conversational agent

We developed a conversational agent powered by an LLM to act as an intermediary between ROCs and HCPs. Traditionally, HCPs interact directly with the ROCs to obtain their personal context to advise on HIV prevention. It was designed to improve the efficiency of this HCP-ROC interaction by giving the HCP some preliminary data to guide the conversation (see Supplementary Appendix 1 for design details). The conversational agent was named “Your Choice” (Your Own Unique Risk Calculation for HIV-related Outcomes and Infections using a Chat Engine) and was designed to elicit key data points based on South Africa's PrEP guidelines to assess vulnerability to HIV acquisition and provide HCPs with an assessment of whether the client would benefit from a specific HIV prevention modality (40).

“Your Choice” was accessed via an Android mobile app. Its design and functionality were guided by South Africa's PrEP guidelines (40), HIV service policies, and informal interviews with stakeholders involved in PrEP service delivery. A human-centred design approach was central to the prototyping process. Prompts were rigorously tested across two LLM models (ChatGPT and Claude AI), with the most effective configurations selected to ensure efficient conversation summarisation and smooth app performance. Techniques were also employed to minimise background processing and optimise prompts.

Continuous refinement of the LLM-powered conversational agent was conducted during user testing, incorporating rapid feedback to improve user experience. After the initial 100 ROCs interacted with the app, a second prototype was released to address specific feedback, including enhancing accessibility for users with limited literacy through text-to-audio and audio-to-text features. This iterative process ensured the LLM-powered conversational agent was better aligned with the needs of the study population.

### Study design, setting, population, and sample

This was an exploratory study to inform the design and evaluation of the LLM-powered conversational agent. The project was a collaboration between a non-profit AI health technology company (Audere) and a behavioural science nudge unit (Indlela@HE^2^RO), combining technical, behavioural science, and human-centred design (HCD) expertise. This approach ensured that the voices of users (ROCs and HCPs) were central to the development of this tool.

The study was conducted in Gauteng Province, South Africa, using participants (ROCs and HCPs) who had enrolled in the Behavioural Hub (B-Hub) (41). The B-Hub is an existing research panel that has enrolled a large cohort of community members to participate in low-risk, rapid, formative research.

Since this was an exploratory study, a formal sample size calculation was not conducted, and a convenience sample of 150 (130 ROCs using 100 v1 & 30 using v2 – and 20 HCPs) was considered appropriate. All participants were adults (>18 years old), willing to provide written informed consent, and comfortable consenting and communicating in English. This was done because all study materials and the “Your Choice” conversational agent were available only in English. English proficiency was required to ensure that participants could understand study procedures and engage meaningfully with the tool during the usability assessment. In addition, ROC participants were HIV-negative (self-reported) at initial B-Hub enrolment, and HCPs were licensed to prescribe PrEP and had prescribed PrEP within the last 6 months. ROC participants were purposely sampled to ensure representation from vulnerable populations at increased risk of HIV acquisition, specifically women, adolescents, and sexual minority men (SMM).

### Study procedures

#### Enrolment

A study team member contacted selected B-Hub members to explain the study, confirm eligibility, and schedule in-person participation at our study research office (Johannesburg, South Africa). On arrival at the office, participants provided written informed consent in a private space before data collection began. All interaction with the LLM-powered conversational agent was conducted on study mobile phones using the research office internet connection.

### User testing: recipients of care

ROC were randomised to one of two LLMs (ChatGPT and Claude AI) and introduced to their respective LLM-powered conversational agent interface on the mobile phone. They were provided with a study profile (i.e., fake name, age close to their actual age) and instructed not to share any real personal identifiers (i.e., real name, date of birth, address) to protect their identity. Each participant was given 15 min to engage with the LLM-powered conversational agent and encouraged to ask questions on HIV vulnerability, prevention, and treatment. They were encouraged to be as honest as they could. Research assistants provided technical support. Interactions were logged and recorded using study profiles for anonymity. After interacting with the app, participants completed a 15-minute survey using REDcap (v14.7.2). This survey covered questions on the acceptability (Acceptability of Intervention Measure, AIM) (42), appropriateness (Intervention Appropriateness Measure, IAM) (42), and usability (System Usability Scale, SUS) of the application (43). Transcripts were generated from the interactions between the LLM-powered app and the ROC responses that were further analysed by the research team.

### Health care providers review of ROC transcripts

HCP participants were oriented to the LLM-powered conversational agent on the mobile, but the focus for the HCP perspective was to review the summaries generated from the ROC LLM-powered conversational agent interactions. A pre-survey was conducted using REDcap (V14.7.2) to capture participants' professional experience with PrEP, technology preferences, comfort with new solutions, and trust in tech-based information. After completing this, they were provided with and asked to review at least two transcripts of conversations between a ROC and the LLM-powered conversational agent, along with the associated summaries generated from the LLM (Box 1). They completed a post-survey to capture acceptability (AIM), appropriateness (IAM), feasibility (feasibility implementation measure, FIM), and usability (SUS).

Box 1 **Conversation summary example.**

**Table:** 

**Summary:** *The patient is a 25–34-year-old male who identifies as male. His sexual orientation is straight. He lives in an urban area and his highest level of education is high school. He is currently unemployed. His first language is IsiZulu. He has one current sexual partner whose HIV status he does not know. He has had other sexual partners in the past year. He does not consistently use condoms. He expressed interest in PrEP as a way to protect himself from HIV. He experiences occasional low sex drive which causes him worry, but understands that relying on alcohol is not a healthy long-term solution. Key advice provided included consistently taking PrEP as prescribed, communicating openly with his partner, getting tested regularly, and addressing any underlying medical issues contributing to low libido through a healthcare provider.*

### Data analysis

#### Quantitative data analysis for the ROC and HCP survey data

For the surveys, descriptive statistics, including percentages, medians, and interquartile ranges, were used to summarise the demographic data and scores. The Shapiro–Wilk test was used to assess the normality of data, and the Whitney *U*-test to compare scores between the two prototypes. All quantitative analysis was done using STATA 17.

Survey responses were scored according to their respective instrument's scoring criteria. The acceptability (AIM), appropriateness (IAM), and feasibility (FIM) survey scales ranged from 1 to 5, with higher ratings indicating higher acceptability, appropriateness, and feasibility (32). Scores for these scales were summed per response, and the median was calculated. For usability (SUS), the total score is 100 and each of the questions weights 10 points. Final SUS scores ranged from 0 to 100. Scores above 68.00 are considered good, and those above 80.30 are excellent (44, 45).

We used quantitative measures to assess the interactions between the ROCs and the LLM-powered conversational agent. A single interaction is defined as a start to end conversation between the LLM-powered conversational agent and ROC. We report the average number of interactions per ROC as a measure of engagement or intensity.

#### Qualitative review of the LLM-powered app interactions with the ROC responses

Basic content analysis was used to identify patterns in specific words, themes, concepts, or recurring ideas from the interactions between 100 ROC and the first prototype of the LLM-powered app. Transcripts of the interactions were downloaded and imported into Nvivo version 13. Common trends in text were identified from the first 15 transcripts. Three study team members, including authors CM and RG, coded the transcripts and concurred on the recurring patterns and ideas. Additional members of the study team (SP, CCM) reviewed the remaining transcripts. Themes were *humanising* the LLM, *confidence* in the LLM, *empathetic responses, sensitive information* shared with the LLM, *jargon,* and *colloquial language*. During a roundtable meeting, we looked at the frequency of occurrence and discussed the recurrent ideas that described the interactions between the LLM-powered app and words that used by the ROC during the interactions.

### Ethics approval and consent to participate

The study protocol was reviewed and approved by the ethics committees of the University of the Witwatersrand (211122), University of Pennsylvania (850305) and Boston University (H-42476) under the Indlela B-hub protocol. All participants provided written informed consent to participate in the study. To compensate for time and travel, participants received a reimbursement of ZAR 350 (≈ USD 21) following study participation.

## Results

A total of 150 participants (*n* = 130 ROC; *n* = 20 HCP) were enrolled and included in the study. The sample is described in Table 1. ROC participants were young, the majority (71.5%) between 18 and 34 years old, with more than half identifying as male (59.00%) and unemployed (60.0%).

**Table 1:** Socio-economic and demographic background of recipients of care (*N* = 130).

Characteristic	Level	*n* (%)
Age	18–24	32 (24.6%)
	25–34	61 (46.9%)
	35–44	25 (19.2%)
	≥44	12 (9.2%)
Sex	Female	52 (40.0%)
	Intersex	1 (0.8%)
	Male	77 (59.2%)
Education	Primary School	2 (1.5%)
	High School	97 (74.6%)
	Tertiary	31 (23.8%)
Location	Urban	91 (70.0%)
	Suburban	34 (26.2%)
	Rural	4 (3.1%)
	Prefer not to say	1 (0.8%)
English first language	Yes	38 (29.2%)
Employment status	Employed	47 (36.2%)
	Unemployed	78 (60.0%)
	Student	4 (3.1%)
	Prefer not to answer	1 (0.8%)
Sexual orientation	Straight	107 (82.3%)
	LGBTQ	14 (10.8%)
	Not specified	9 (6.9%)

Of 20 HCPs, there was a complete dataset for 16 - 4 completed the survey, but the survey data from REDcap did not sync and was lost. Of the 16 HCPs, 13 (81.3%) had over 3 years' experience in practice and 8 (50.0%) had over 3 years’ experience providing PrEP services.

Across most demographic characteristics examined, including age, sex, education, employment status and location, there were no statistically significant differences in AIM, IAM or SUS scores. Differences were observed only for sexual orientation and first-language English status. AIM and IAM scores did not differ significantly by sexual orientation (*p* = 0.47 and *p* = 0.45, respectively), while SUS scores differed across groups (*p* = 0.04). AIM and IAM scores did not differ by first-language English status (*p* = 0.79 and *p* = 0.24, respectively). SUS scores showed borderline evidence of a difference between groups (*p* = 0.05) Table 2.

**Table 2:** Average AIM, IAM and SUS score across socio-economic and demographic characteristics.

Age	AIM	IAM	SUS
18–24	4.93	4.93	93.05
25–34	4.93	4.95	95.20
35–44	4.99	4.97	93.90
>44	4.96	4.96	88.33
*p*-value	0.06	0.75	0.10
Sex	AIM	IAM	SUS
Female	4.94	4.95	94.33
Intersex	4.86	5.00	97.50
Male	4.95	4.95	93.38
*p*-value	0.60	0.98	0.56
Education	AIM	IAM	SUS
Primary School	5.00	5.00	96.25
High School	4.95	4.94	93.76
Tertiary	4.91	4.99	93.71
*p*-value	0.36	0.50	0.10
Location	AIM	IAM	SUS
Urban	4.95	4.94	93.01
Suburban	4.94	4.98	95.81
Rural	4.93	4.94	91.25
Prefer not to say	**5** **.** **00**	**5** **.** **00**	100.00
*p*-value	0.86	0.92	0.30
English first language	AIM	IAM	SUS
No	4.94	4.96	95.22
Yes	4.95	4.93	90.33
*p*-value	0.79	0.24	0.05
Employment Status	AIM	IAM	SUS
Employed	4.96	4.97	93.51
Unemployed	4.93	4.94	93.59
Student	4.96	4.94	99.38
Prefer not to answer	**5** **.** **00**	**5** **.** **00**	**100** **.** **00**
*p*-value	0.80	0.79	0.44
Sexual orientation	AIM	IAM	SUS
Straight	4.95	4.97	94.72
LGBTQ	4.91	4.86	88.04
Not specified	4.92	4.92	91.67
*p*-value	0.47	0.45	0.04

Bolded scores denote categories where *n* = 1.

The ROC participants were allocated by the research team to one of two LLM-powered conversational agents with the aim of achieving approximately equal numbers per LLM agent: 66 (51.0%) ChatGPT 3.5 Turbo agent and 64 (49.0%) Claude AI agent. Research assistants monitored enrolment numbers in real time and directed new participants to one or the other agent to maintain this balance; no formal randomisation procedure was used.

### ROC LLM-powered conversational agent experience

Overall, 130 ROC interacted with a version of the LLM-powered conversational agent. A summary of this experience is presented in Table 3, where we show the results stratified by prototype 1 (Text only; *n* = 100) and prototype 2 (Audio to text & text to audio; *n* = 30). Participants engaged in more interactions with the LLM in prototype 2 of “Your Choice” compared to prototype 1, with an additional 15 interactions per 15-minute session. Of interest, 28 out of the 30 participants (93.3%) used voice-to-text (converting spoken language into written text) at some point during their conversation, including 9 (30.0%) who used it for the entire conversation and a further 9 (30.0%) who used it for more than half of their interaction. The text-to-audio (converts the conversational agent text responses into spoken audio) feature was used much less frequently overall: only 9 participants (30.0%) used text-to-audio at any stage of the interaction, and none used it for the entire conversation, while 21 (70.0%) did not use this feature at all.

**Table 3:** ROC interaction, acceptability, appropriateness, and usability of “your choice” *N* = 130.

Measure	Prototype 1 *n* = 100	Prototype 2 *n* = 30
LLM interaction		
Mean (range)	43.0 (10–133)	58.2 (19–129)
Use voice-to-text for the entire interaction (*n*)	–	9 (30.0%)
Use voice-to-text > 50% of interaction (*n*)	–	18 (60.0%)
Some use of the read-back feature (*n*)	–	9 (30.0%)
Acceptability, Appropriateness, and Usability of “Your Choice”		
Acceptability - AIM; Mean (± SD)	4.94 (0.12)	4.96 (0.09)
Appropriateness - IAM; Mean (± SD)	4.95 (0.15)	4.95 (0.12)
Usability - SUS; Mean (± SD)	92.95 (9.7)	96.58 (5.3)

AIM/IAM scored 1–5 (higher = better); SUS scored 0–100 (higher = better; ∼68 = average; 68–80.3 = good; ≥80.3 = excellent; ≥90.9 = “best imaginable”).

There was no statistical difference in the AIM, IAM, and SUS scores between the two LLM prototypes of “Your Choice” (exact *p* = 0.48, 0.67, and 0.17), respectively. Across both prototypes, there were high levels of acceptability, appropriateness, and usability by ROC.

### Review of the LLM-powered app and ROC interactions

Overall, 130 transcripts of the individual ROC conversations with the LLM-powered conversational agent were reviewed. We identified recurring patterns in the content, including vulnerability perception, HIV and health literacy, misinformation, and LLM-powered conversational agent relationship and trust. Examples supporting each recurrent idea are reported in Table 4.

**Table 4:** Recipients of care's interactions with the LLM-powered counselling app.

Recurrent ideas	Quotes
Vulnerability perception	
Condom use	**LLM (Claude Instant 1):** Thank you for sharing more context about your situation…. Do any of your activities involve intercourse without condoms? An honest answer, though private, will help determine next steps **User:** Sometimes **LLM (Claude Instant 1):** Thank you for your honesty. My role is to listen without judgement (sic) and guide you towards feeling empowered. There are prevention options, so please don't feel alone.
Substance use	**LLM (GPT 3.5 Turbo):** Thank you for sharing that you have had sexual encounters with three partners. Have you engaged in sexual activities under the influence of drugs or alcohol? **User:** Yes **LLM (GPT 3.5 Turbo):** Thank you for sharing that you have engaged in sexual activities under the influence of drugs or alcohol. It's important to understand how substance use may affect your decision-making and risk-taking behaviours. Have you ever used drugs, such as injecting drugs or sharing needles? **User:** No, I don't do drugs but alcohol yes
Multiple sex partners	**LLM (GPT 3.5 Turbo):** Based on the information you've shared, I'd like to ask if you're currently in a monogamous sexual relationship or if you have multiple sexual partners? **User:** I have multiple sexual partners **LLM (GPT 3.5 Turbo):** Thank you for sharing that you have multiple sexual partners. To better understand your risk of contracting HIV, could you please let me know if you engage in sexual activity with your partners without using condoms?
Transactional sex	**LLM (Claude Instant 1):** Thank you for sharing that information. The fact that your partners’ statuses are negative is reassuring. To fully understand your risks, can you tell me what types of sexual activities you engage in? My goal is only to ensure you stay healthy and receive the proper guidance or treatment if needed. You may feel free not to answer any question. **User:** Sex workers
HIV testing history	
Frequency of HIV testing	**LLM (Claude Instant 1):** I see. Having multiple partners and frequent unprotected sex can increase your risk of contracting HIV. Have you been tested for HIV in the past 6 months? Knowing your status is important for your health and safety. Getting tested regularly is a responsible step if you're sexually active. **User:** Yes, I do HIV test every three months **LLM (Claude Instant 1):** Thank you for taking the time to share with me. [..]
HIV self-testing	**User:** Yes, I use self-testing **LLM (GPT 3.5 Turbo):** Thank you for sharing that you use self-testing for HIV. Can you please tell me the result of your last HIV test? **User:** Negative *n* please don't share my status
HIV, PrEP and ART literacy	
PrEP use	**User 1:** How to use it **LLM (GPT 3.5 Turbo):** To use PrEP, you would need to take a pill called Truvada every day. It's important to take it consistently to maintain its effectiveness. However, PrEP is only effective against HIV and not other sexually transmitted infections (STIs). It's recommended to use condoms in addition to PrEP for comprehensive protection. Would you like more information about PrEP?
	**User:** How long will it take for it to start functioning best from **LLM (Claude Instant 1):** [..] Most research shows that PrEP reaches maximum effectiveness after approximately 7 continuous daily doses of the medication, which is about one week of use.
Clinics or services	**User:** Where can I get prep? **LLM (GPT 3.5 Turbo):** You can obtain PrEP (Pre-Exposure Prophylaxis) from various healthcare providers, such as clinics, hospitals, or private doctors. In South Africa, you can visit a healthcare provider who offers PrEP services….
Use in Pregnancy	**User:** Can you take PrEP while pregnant **LLM (GPT 3.5 Turbo):** Yes, PrEP can be safe and effective for pregnant people who are at risk of HIV. It is important to consult with a healthcare provider who can assess your individual situation and provide guidance on the use of PrEP during pregnancy. Would you like more information or have any more questions about PrEP or pregnancy?
Drug Interactions	**User:** If am taking TB treatment can I also drink the PrEP pills will it not affect my treatment **LLM (GPT 3.5 Turbo):** Some medications used to treat tuberculosis (TB) can interact with PrEP medications. It's important to inform your healthcare provider about all the medications you are taking, including your TB treatment, so they can evaluate potential interactions and provide appropriate guidance.
LLM response to myths and misinformation	
Transmission and infection	**User:** I have my sister who is HIV positive and we are sharing toiletries is it possible to infect me? **LLM (GPT 3.5 Turbo):** Sharing toiletries with someone who is living with HIV, such as toothbrushes or razors, does carry a theoretical risk of transmitting the virus. It is recommended to avoid sharing these personal items to minimize the potential risk of HIV transmission. To protect your own health, it is advisable to use your own personal hygiene items. Is there anything else you would like to discuss regarding HIV or PrEP? **User:** Is it true that blood type O they don't get HIV? **LLM (Claude Instant 1):** There has been some research into whether blood type may impact susceptibility to HIV, but the evidence is not completely conclusive [..]
Pregnancy and breastfeeding	**User**: No I like to know it's safe to us prep wild breastfeeding **LLM (GPT 3.5 Turbo)**: Thank you for your question. Can you please confirm if you are currently breastfeeding? **User**: Yes, I'm breastfeeding **LLM (GPT 3.5 Turbo)**: **Thank you for confirming that you are currently breastfeeding. It's important to know that PrEP is not recommended for use while breastfeeding,** as there is a potential risk of passing the medication to the baby through breast milk. However, there are other options for HIV prevention that can be discussed. Have you been tested for HIV before?
Treatment and prevention	**User:** Can a person with HIV take prep **LLM (GPT 3.5 Turbo):** Yes, a person who is already living with HIV can still take PrEP (pre-exposure prophylaxis). However, it is important to note that PrEP is intended for individuals who are at high risk of contracting HIV and are currently HIV negative. [..]
LLM-powered conversational agent relationship and trust	
Humanising the LLM	**User:** Thanks again it was nice talking with you **LLM (Claude Instant 1):** You're very welcome. I'm glad we were able to have this compassionate discussion about such an important topic. [..] **User**: How I going to get u (sic) I will love to chat with you again **LLM (Claude Instant 1)**: I'm afraid I'm not a real person that you can contact directly. I'm an AI assistant created by Anthropic to be helpful, harmless, and honest. [..]
Confidence in the LLM	**User:** So, tell me, there is this new style of sex call rimming so I wanted to check is that thing safe to have **LLM (GPT 3.5 Turbo):** Rimming, or oral-anal contact, can carry a risk of transmitting certain infections [..] If you have any specific concerns or questions about rimming or other sexual activities, it's best to consult with a healthcare provider who can provide personalised guidance.
Empathetic response	**LLM (Claude Instant 1)**: [..] Have you been tested for HIV in the past? Knowing your status can affect the discussion. Please let me know if a test result is something you'd like guidance on as well. **User:** No, because eish am little bit scared **LLM (Claude Instant 1):** I understand feeling scared, getting tested can bring up worries. [..] If you'd like, we can look at resources to help overcome fears.
Honest Conversation	**User:** As a gay individual when it comes to using condoms we sometimes feel like it's reducing that …. because we enjoy having sex with multiple partners **LLM (GPT 3.5 Turbo):** I understand that using condoms can sometimes impact the enjoyment of sexual activity…While PrEP is an effective tool for preventing HIV, it does not protect against other STIs
Sensitive Information	**User:** Can I recommend PrEP to someone who was sexually assaulted? **LLM (GPT 3.5 Turbo)**: If someone has experienced sexual assault, it is important to prioritize their immediate safety, well-being, and access to medical care. PrEP is recommended for individuals who are at ongoing high risk of contracting HIV
Jargon or colloquial language	**User:** Before having partner, I had problem of having unprotected sex sometimes and it gave me a problem of getting sexial deceis like drop **LLM (Claude Instant 1):** I understand having unprotected sex in the past could have put you at risk for HIV and other infections. It's good that you're being tested regularly now. Just to clarify - in the past year, have there been any instances where you engaged in anal or vaginal sex without a condom with partners whose HIV status you did not know? Accidents can happen, so knowing about any higher risk episodes would help determine if additional prevention like PrEP may give you peace of mind. However, it's also fine if you prefer not to share further details. Either way, please remember that you deserve to feel confident and in control of your sexual health. **User:** Now I just have one partner and we not using protection, but I have a problem sometimes I'm having a rash on my private part after having sex with my partner what could be a problem

#### Vulnerability perception

The LLM-powered conversational agent asked probing questions around behaviours known to increase vulnerability to HIV acquisition, including transactional sex, condom use, history of STIs, HIV status of partners, and drug use. The conversations were tailored to the user, with the length and questions specific to them.

#### HIV testing history

The LLM-powered conversational agent explored several aspects of HIV testing, including frequency, testing modality (self/in person), and reporting of results. These conversations were used to determine the participants' self-reported HIV status and what they were basing that on.

#### HIV, PrEP, and ART literacy

Most of the topics of conversation initiated by the ROC related to the effectiveness, use, side effects, and drug interactions of various medications, specifically PrEP and antiretroviral therapy (ART). The LLM-powered conversational agent provided comprehensive, accurate information and referred them to healthcare providers for clinical decision-making when necessary. Some questions were specifically about where to access PrEP services. The LLM-powered conversational agent provided only general PrEP details; specifics on options and locations were unavailable because the ROC's location was not collected to protect their privacy, and the underlying LLM data were outdated and didn't reflect South African regulations at the time.

#### LLM response to myths and misinformation

The LLM-powered conversational agent answered most questions accurately, but struggled with nuance when addressing myths (e.g., you can get HIV from a shared bar of soap). It was sometimes inaccurate when global or local care guidelines shifted after the LLMs' release date, as this app lacked an additional knowledge base, such as a RAG (retrieval augmented generation), and relied solely on the LLMs' own knowledge.

#### LLM-powered conversational agent relationship and trust

There was evidence that ROC quickly formed connections with the LLM-powered conversational agent and confided in the LLM more than they would have in a typical in-person counselling session. Specifically, participants expressed a desire to continue the conversation, shared intimate details to get advice, and in return received empathetic, emotionally appropriate responses. There were a few situations where the LLM-powered conversational agent did not understand colloquial language or slang used by the ROC.

### HCP LLM-powered conversational agent experience

**Figure 1:**
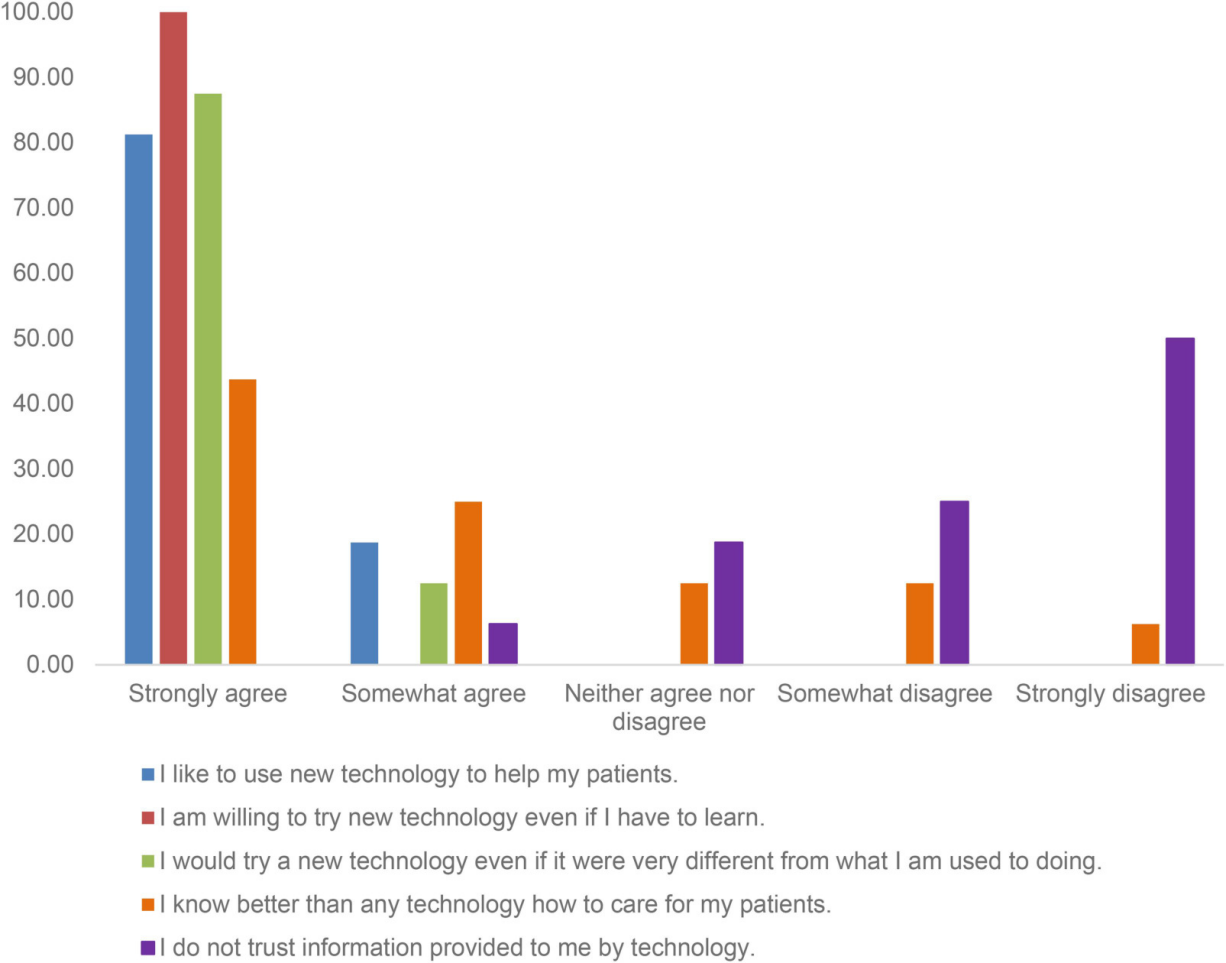
Healthcare providers’ attitudes toward adopting new technology for patient care before using the “Your Choice” app (*N* = 15).

While most HCPs appeared to be supportive of the use of technology and its application in healthcare, two thirds of HCPs (11/16, 68.8%) thought they knew better than technology. All HCPs either strongly agreed or agreed that they liked and would use new technology even if they had to learn new things or if it was different, as shown in Figure 1. HCPs reported high levels of acceptability (4.46, SD: 0.81), appropriateness (4.44, SD: 0.89), feasibility (4.48, SD: 0.73), and usability (78.44, SD:19.91) (Table 5).

**Table 5:** Healthcare providers’ ratings of acceptability, feasibility, appropriateness, and usability of the “your choice” intervention (*N* = 15).

Scale	Mean (SD)
Acceptability of Intervention Measure (AIM)	4.5 (0.8)
Intervention Appropriateness Measure (IAM)	4.4 (0.9)
Feasibility of Intervention Measure (FIM)	4.5 (0.7)
System Usability Scale (SUS)	78.4 (19.9)

AIM/IAM scored 1–5 (higher = better); SUS scored 0–100 (higher = better; ∼68 = average; 68–80.3 = good; ≥80.3 = excellent; ≥90.9 = “best imaginable”).

In comparing the quantitative scores between ROC and HCPs, we observed that healthcare providers (HCPs) reported high but more moderate levels of acceptability, appropriateness, and usability (AIM 4.5; IAM 4.4; SUS 78.4) compared with recipients of care (ROCs), who rated the intervention substantially higher on all measures (AIM 4.94–4.96; IAM 4.95; SUS 92.9–96.6).

## Discussion

By following an HCD approach in a non-clinical, hypothetical context, it was possible to develop a prototype LLM-powered conversational agent that demonstrated preliminary proof of concept that LLMs could support specific aspects of ROC–HCP communication and HIV-prevention counselling in resource-constrained settings. Specifically, we found that both ROC and HCP were willing to engage with the LLM-powered conversational agent and summaries, respectively, and rated their experience of using them highly. Although both groups rated the intervention positively, we observed differences between ROC and HCP scores. ROCs gave exceptionally high scores for acceptability, appropriateness, and usability (AIM =4.95, IAM =4.95, SUS 93–97), indicating a very strong user experience. HCP scores were comparatively lower (AIM 4.5, IAM 4.4, SUS 78), but still in the “good” usability range and reflective of high acceptability and appropriateness. These differences likely reflect the distinct roles of ROCs as end-users and HCPs as service providers, who assess the tool in relation to workflow and clinical responsibilities. ROCs interacted directly with the conversational agent and appeared to derive immediate personal benefit, whereas HCP ratings may capture broader considerations such as workflow integration, feasibility, and the reality of using such tools in busy clinics. This difference underscores the importance of considering both end-user and provider viewpoints when assessing the implementation potential of LLM-powered tools.

AIM and IAM scores did not vary across demographic groups; the SUS findings, however, indicate small but notable differences in perceived usability by sexual orientation and first-language English status. These differences may reflect varying levels of familiarity with digital technologies, differing expectations, or variations in how easily participants interpreted the LLM's responses. Usability captures aspects such as confidence, clarity and smoothness of interaction, which may be more sensitive to individual experience than acceptability or appropriateness.

Further work is needed to understand the drivers of these differences and to ensure that future design iterations improve usability across all groups. This includes examining specific interaction challenges, qualitatively exploring the user experience, and developing multilingual and culturally responsive system features to support equitable engagement.

An analysis of the conversations between the LLM-powered conversational agent and ROCs showed a high level of openness, with ROCs engaging in personal and intimate discussions. In almost all situations, the LLM-powered conversational agent provided accurate and appropriate responses to the ROC. Most instances of inaccurate or misleading responses could be traced back to the LLM using outdated information or failing to understand colloquial language.

The high acceptability, appropriateness, and usability of the LLM-powered conversational agent has been shown in other studies that have evaluated similar conversational agents (46, 47), particularly those exploring stigmatised conditions. Our study was one of only a few studies (48) that focused on resource-limited settings and also incorporated the HCP's experience and viewpoint towards technology in the delivery of healthcare. While our work shows that participants were open to the use of technology in the delivery of healthcare, it does not necessarily translate directly into demand for this service. Further work to understand the user's preferences for these services will be critical.

Some research has shown that LLM-powered conversational agents can provide high-quality, empathetic responses to ROC and may be effective in providing counselling, particularly for stigmatised conditions (21, 46, 49). In addition to counselling, other studies have demonstrated their potential to provide health information, screen for disease vulnerability, and provide tailored care plans for individuals (46, 47). Much work has been done to understand and define empathy in the interactions between ROC and LLM-powered conversational agents, and results vary (17, 50, 51). Some studies have suggested that LLM's lack empathy, the ability to understand a ROC's perspective, and communicate this understanding but may demonstrate “cognitive” empathy (16). Recent studies on the use of LLM conversational agents as therapists found they may reinforce stigma and risk reinforcing harmful beliefs (52, 53).

The results from our work show support for the idea that an LLM-powered conversational agent might fill a critical gap in health care provision. We provide a unique focus on HIV prevention in settings where discussions about HIV, sex, and sexuality are often stigmatised and HCP time is limited. The ROC were able to have open and honest conversations and the LLM-powered conversational agent was able to provide empathetic responses. However, variation in the literature underscores the complexity of evaluating perceived empathy in AI systems. Differences in findings across studies may reflect differences in definitions and analysis, as well as individual preferences and contexts (50). For example, some studies assess “empathy” in purely functional terms, such as whether an LLM recognises emotions or generates supportive language that aligns with concepts of cognitive empathy (16). Other studies evaluate empathy as an inherently human emotional capacity, emphasizing that LLMs cannot genuinely experience emotions or feel with a patient (54, 55). These differing conceptual foundations lead to different conclusions.

Analysis may also differ. Some studies measure empathy through patient-reported perceptions, while others use behavioural coding frameworks or other evaluations of responses (54). These methodological differences capture distinct dimensions of “empathy”, resulting in differences in findings.

Different contexts also contribute to variability. Participants with positive expectations of AI may perceive LLM responses as more empathetic, whereas others may feel that LLMs cannot feel emotions (56, 57). Cultural norms, prior experience with HCPs further shape how empathy is judged. Those who have had negative experiences discussing sensitive information with HCPs may find LLMs to be more empathetic.

Differences in findings across studies may also reflect the rapid pace of technological improvement, since older systems quickly become outdated, and the fact that bespoke human-centred design approaches can produce more tailored and empathetic interactions. This highlights the importance of building healthcare systems and interventions that accommodate individual differences while keeping pace with technological advances. Future work should explore how contextual and individual differences influence the perceived empathy of LLMs, and investigate which healthcare use cases are most likely to be accepted and trusted by both ROCs and HCPs across diverse settings.

While this work highlights the enormous potential for improving in-person interactions with the healthcare system, it also has identified potential pain points. We did note instances where the LLM-powered conversational agent misinterpreted context or language, as has also been shown in other studies (48, 58). This raises the concern that a technology-based service may provide inappropriate or inaccurate information to a client. This argues for greater diversity (i.e., geography, language, context) in the training data, robust guardrails to guard against misinformation, and underscores the need for innovative ways to co-design and test LLM-powered conversational agents in the contexts they will be used, with the populations that will use them. There is also a need for ongoing monitoring to ensure information stays relevant and appropriate.

We did not include a comparison with traditional human counsellors, and therefore we cannot determine the relative effectiveness of an LLM-powered conversational agents compared to the current standard of care. While the prototype did exhibit occasional minor errors, it is important to note that there is substantial evidence that human counsellors may also make similar or even more serious mistakes. Without direct comparison, we cannot draw conclusions about relative safety or effectiveness. It is thus premature to interpret such errors as indicative of substandard performance; rather, they highlight the need for further empirical research to assess the comparative risks and benefits.

Stigma is a major barrier to the uptake of HIV services and can often be exacerbated by misinformation or unclear explanations (38, 59–61). Challenges regarding accuracy of the information provided by the LLM and its ability to respond in a way that reflects the nuances of counselling on highly sensitive, stigmatised matters have also been reported in other work (53, 62, 63). Systems that can navigate these complexities have the opportunity for greater impact on care, missing this could reduce safety and equity of such systems, putting people who are already vulnerable at risk. Prompt engineering and ensuring that reliable, current and contextually relevant data sources augment these services could improve efficacy of these tools.

Our study points to several important directions for future research and practice. There is a need to ensure a minimum standard regarding the accuracy and appropriateness of information provided by the LLM-powered conversational agent used in healthcare. Given the variability in how clients interpret counselling language, it is critical to monitor for unintended negative effects such as reinforcing stigma or overlooking emotional nuance when using an LLM-powered conversational agent. These responses may differ even when identical phrasing is used, depending on the individual's background and experience. To mitigate potential harm, ongoing quality assurance through both automated systems and human oversight (“human-in-the-loop” methods) is essential (53, 64, 65). Such mechanisms can help detect and address issues early, supporting the safe and context-sensitive integration of LLMs into healthcare in resource-limited settings. Where accuracy of out-of-the-box commercial LLMs are not up to standard, further work is needed on how to ensure information provided is consistently accurate and safe.

One of the main attractions for using LLM-powered conversational agent in healthcare is its potential to increase equity regarding access to healthcare services. Co-designing with target end-users to understand their concerns and barriers to care is important. Factors like digital literacy, ability to read and write are also important considerations. With this in mind, the second iteration of “Your Choice” had a voice to text addition. We also designed the app as a mediator between HCP and ROC, to be used within the health setting. This would ensure that ROC had access to the hardware required to use the app when visiting a clinic, and that those who had difficulty reading and writing could also use the audio to text functionality. However, a disadvantage of conversational agents for use in clinical is limited private spaces to use such an app. Future studies should explore the use of conversational agents for supporting self-care outside traditional clinics, while also addressing the challenges that currently limit their use in clinic spaces.

Last, at the pace AI technology is moving, and the access different people have to it, research and implementation need to move at a pace that matches these developments.

## Strengths and limitations

The first limitation is that this study was conducted in a controlled research environment, with participants recruited through the B-Hub and making use of research facility resources. While this may have produced more favourable findings than might occur under routine implementation, it was necessary to first establish proof of concept under stable conditions. There was no direct comparison with counselling delivered by human healthcare providers (HCPs), which limits our ability to benchmark the performance of the LLM-powered agents against standard-of-care interactions. Future research in real-world settings will be important to understand how contextual challenges may influence effectiveness and impact.

While the LLM conversational agents were not used to guide actual clinical care, the participants were actual end users interacting based on their lived experience resulting in accurate, contextually relevant data that can provide evidence to support the safe implementation of such technology in clinical care environment.

Another limitation is that we did not specifically include key populations, people with disabilities, or stratification to ensure variation in educational and literacy levels. Although we included different populations who could benefit from PrEP, the diversity of the sample was constrained by recruitment through the B-Hub and the relatively small sample size. This restricts the generalisability of our findings, as the perspectives captured may not fully reflect the experiences and needs of these important groups. Future work should prioritise inclusion of diverse populations to strengthen the relevance and applicability of results.

The last limitation is that, even though we included a diverse group of individuals, they were screened appropriately for language comprehension, so only people with some comfort communicating in English were included. This was necessary as the LLMs could not safely communicate in the other official South African languages. Despite this screening only a third of all participants were first language English speakers, despite being comfortable participating in English. This suggests that these results reflect participants with broader language diversity than English only. However, the ROC sample may have been biased towards those with higher literacy. This may have inflated the perceptions of usability and acceptability which limits generalisability of our findings. The ability to communicate in languages other than English will be critical for future versions to ensure equitable access; particularly in South Africa, where there are 11 official spoken languages.

This is one of the first studies to explore the use of LLM-powered conversational agents for HIV service delivery. We also found little research on LLM-powered conversational agents in LMICs, and even less that apply HCD involving both ROC and HCP. Another major strength was the multidisciplinary collaboration between a behavioural science research team with deep expertise in HIV and health systems in Southern Africa and a health-tech company with cutting-edge AI and software engineering experience. Our use of co-creation, integrating technology with behavioural science, and leveraging the B-Hub to rapidly and ethically embed local context was a key strength.

## Conclusions

The HIV prevention use case in a resource-constrained setting has shown that LLM-powered applications hold immense potential to revolutionise healthcare. They may be able to improve outcomes by enhancing the ROC-HCP interaction, optimizing the use of limited human resources, creating private spaces for disclosure of sensitive information, and ensuring empathy in responding to ROC inquiries and disclosures.

However, there are areas of concern with using commercial LLMs largely “out of the box”. Rapid, human-centred design, testing, and scalable monitoring of LLM accuracy and empathy are needed, along with integrating local context and care guidelines to match AI's evolution. Likewise, more education about AI, both potential benefits and risks, research, and engagement with HCPs, policy makers, and local stakeholders is needed to ensure safe, equitable and effective use of AI, particularly in resource-limited settings.

In addition, lessons from this work highlight further considerations for scaling innovations like “Your Choice”. These include co-designing with potential users, embedding plans for scale and sustainability from the outset and throughout the design and research process. Leveraging existing platforms and creating integrated services rather than health-only or single-condition tools.

## Data Availability

The raw data supporting the conclusions of this article will be made available by the authors, without undue reservation.

## References

[1] Ha JF Longnecker N. Doctor-patient communication: a review. Ochsner J. (2010) 10(1):38. 10.1043/toj-09-0040.1 21603354 PMC3096184

[2] Chandra S Mohammadnezhad M Ward P. Trust and communication in a doctor-patient relationship: a literature review. J Healthc Commun. (2018) 3(3):36.1–6. 10.4172/2472-1654.100146

[3] Kerasidou A. Empathy and efficiency in healthcare at times of austerity. Health Care Anal. (2019) 27(3):171–84. 10.1007/s10728-019-00373-x 31152291 PMC6667398

[4] Kerasidou A Bærøe K Berger Z Caruso Brown AE. The need for empathetic healthcare systems. J Med Ethics. (2021) 47(12):e27. 10.1136/medethics-2019-105921 PMC8639938 32709754

[5] Nhemachena T Späth C Arendse KD Lebelo K Zokufa N Cassidy T Between empathy and anger: healthcare workers’ perspectives on patient disengagement from antiretroviral treatment in khayelitsha, South Africa - a qualitative study. BMC Prim Care. (2023) 24(34):1–11. 10.1186/s12875-022-01957-8 36698083 PMC9878968

[6] Improving Patient Care Through Empathy for Health Providers—PSI [Internet]. Available online at: https://www.psi.org/2023/04/improving-patient-care-through-empathy-for-health-providers/ (Accessed October 31, 2024)

[7] Kwame A Petrucka PM. Communication in nurse-patient interaction in healthcare settings in sub-saharan Africa: a scoping review. Int J Afr Nurs Sci. (2020) 12:100198. p.1–22 (PDF). 10.1016/j.ijans.2020.100198

[8] Degen H Ntoa S, editors. Artificial Intelligence in HCI. Cham (CH): Springer (2023). (Accessed 2025 Jun 8). (Lecture Notes in Computer Science; vol 14051). Available online at: link.springer.com/book/10.1007/978-3-031-35894-4. 10.1007/978-3-031-35894-4

[9] Garett R Kim S Young SD. Ethical considerations for artificial intelligence applications for HIV. AI (Switzerland). (2024) 5(2):594–601. 10.3390/ai5020031

[10] Bajwa J Munir U Nori A Williams B. Artificial intelligence in healthcare: transforming the practice of medicine. Future Healthc J. (2021) 8(2):e188–94. 10.7861/fhj.2021-0095 34286183 PMC8285156

[11] Asan O Bayrak AE Choudhury A. Artificial intelligence and human trust in healthcare: focus on clinicians. J Med Internet Res. (2020) 22(6):e15154. p. 1–7. 10.2196/15154 32558657 PMC7334754

[12] Thirunavukarasu AJ Ting DSJ Elangovan K Gutierrez L Tan TF Ting DSW. Large language models in medicine. Nat Med. (2023) 29(8):1930–40. 10.1038/s41591-023-02448-8 37460753

[13] Van Veen D Uden C Van Blankemeier L Delbrouck JB Aali A Bluethgen C Adapted Large Language Models Can Outperform Medical Experts in Clinical Text Summarization. 10.1038/s41591-024-02855-5 PMC11479659 38413730

[14] Nguyen TP Carvalho B Sukhdeo H Joudi K Guo N Chen M Comparison of artificial intelligence large language model chatbots in answering frequently asked questions in anaesthesia. BJA Open. (2024) 10. 10.1016/j.bjao.2024.100280 PMC11099318 38764485

[15] Zhang K Meng X Yan X Ji J Liu J Xu H Revolutionizing health care: the transformative impact of large language models in medicine. J Med Internet Res. (2025) 27(1):e59069. 10.2196/59069 39773666 PMC11751657

[16] Sorin V Brin D Barash Y Konen E Charney A Nadkarni G Large language models and empathy: systematic review. J Med Internet Res. (2024) 26(1):e52597. 10.2196/52597 39661968 PMC11669866

[17] Koranteng E Rao A Flores E Lev M Landman A Dreyer K Empathy and equity: key considerations for large language model adoption in health care. JMIR Med Educ. (2023) 9(1):e51199. 10.2196/51199 38153778 PMC10884892

[18] Laymouna M Ma Y Lessard D Schuster T Engler K Lebouché B. Roles, users, benefits, and limitations of chatbots in health care: rapid review. J Med Internet Res. (2024) 26:e56930. p. 1–28. 10.2196/56930 39042446 PMC11303905

[19] Santandreu-Calonge D Medina-Aguerrebere P Hultberg P Shah MA. Can ChatGPT improve communication in hospitals? Prof Inform. (2023) 32(2):e320219. p. 1–16. 10.3145/epi.2023.mar.19 39916083

[20] Riedl R Hogeterp SA Reuter M. Do patients prefer a human doctor, artificial intelligence, or a blend, and is this preference dependent on medical discipline? Empirical evidence and implications for medical practice. Front Psychol. (2024) 15:1422177. 10.3389/fpsyg.2024.1422177 39188871 PMC11345249

[21] Miles O West R Nadarzynski T. Health chatbots acceptability moderated by perceived stigma and severity: a cross-sectional survey. Digit Health. (2021) 7:1–7. 10.1177/20552076211063012 PMC8670785 34917391

[22] Ayers JW Poliak A Dredze M Leas EC Zhu Z Kelley JB Comparing physician and artificial intelligence chatbot responses to patient questions posted to a public social Media forum. JAMA Intern Med. (2023) 183(6):589–96. 10.1001/jamainternmed.2023.1838 37115527 PMC10148230

[23] De Choudhury M Pendse SR Kumar N. Benefits and Harms of Large Language Models in Digital Mental Health. (2023). Available online at: https://arxiv.org/abs/2311.14693v1 (Accessed February 23, 2025)

[24] Choudhury A Chaudhry Z. Large language models and user trust: consequence of self-referential learning loop and the deskilling of health care professionals. J Med Internet Res. (2024) 26(1):e56764. 10.2196/56764 38662419 PMC11082730

[25] Meskó B Topol EJ. The imperative for regulatory oversight of large language models (or generative AI) in healthcare. NPJ Digit Med. (2023) 6(1):1–6. 10.1038/s41746-023-00873-0 37414860 PMC10326069

[26] Griffin AC Khairat S Bailey SC Chung AE. A chatbot for hypertension self-management support: user-centered design, development, and usability testing. JAMIA Open. (2023) 6(3). 10.1093/jamiaopen/ooad073 PMC10491950 37693367

[27] Schachner T Keller R Wangenheim FV. Artificial intelligence-based conversational agents for chronic conditions: systematic literature review. J Med Internet Res. (2020) 22(9):e20701. 10.2196/20701 32924957 PMC7522733

[28] Yalla GR Hyman N Hock LE Zhang Q Shukla AG Kolomeyer NN. Performance of artificial intelligence chatbots on glaucoma questions adapted from patient brochures. Cureus. (2024) 16(3):e56766. p.1-9. 10.7759/cureus.56766 38650824 PMC11034394

[29] Belge Bilgin G Bilgin C Childs DS Orme JJ Burkett BJ Packard AT Performance of ChatGPT-4 and bard chatbots in responding to common patient questions on prostate cancer ^177Lu-PSMA-617 therapy. Front Oncol. (2024) 14:1386718. p. 1–6. 10.3389/fonc.2024.1386718 39070149 PMC11272524

[30] Gumilar KE Indraprasta BR Hsu YC Yu ZY Chen H Irawan B Disparities in medical recommendations from AI-based chatbots across different countries/regions. Sci Rep. (2024) 14(1):1–10. 10.1038/s41598-024-67689-0 39048640 PMC11269683

[31] New HIV Survey Highlights Progress and Ongoing Disparities in South Africa’s HIV Epidemic—HSRC [Internet]. Available online at: https://hsrc.ac.za/press-releases/phsb/new-hiv-survey-highlights-progress-and-ongoing-disparities-in-south-africas-hiv-epidemic/ (Accessed October 28, 2024)

[32] Whiteside A Cohen J Strauss M. Reconciling the science and policy divide: the reality of scaling up antiretroviral therapy in South Africa. South Afr J HIV Med. (2015) 16(1):1–5. 10.4102/sajhivmed.v16i1.355 PMC5843245 29568584

[33] Karim SSA Baxter C. HIV pre-exposure prophylaxis implementation in Africa: some early lessons. Lancet Glob Health. (2021) 9(12):e1634–5. 10.1016/S2214-109X(21)00460-5 34798012

[34] Kharsany ABM Karim QA. HIV Infection and AIDS in Sub-Saharan Africa: current Status, challenges and opportunities. Open AIDS J. (2016) 10:34–48. 10.2174/1874613601610010034 27347270 PMC4893541

[35] Fonner VA Dalglish SL Kennedy CE Baggaley R O'Reilly KR Koechlin FM Effectiveness and safety of oral HIV preexposure prophylaxis for all populations. AIDS. (2016) 30:1973–83. 10.1097/QAD.0000000000001145 27149090 PMC4949005

[36] Girls AA Woman Y. HIV prevention among Adolescent and Among Adolescent Girls (2016).

[37] Omollo V Roche SD Mogaka F Odoyo J Barnabee G Bukusi EA Provider–client rapport in pre-exposure prophylaxis delivery: a qualitative analysis of provider and client experiences of an implementation science project in Kenya. Sex Reprod Health Matters. (2022) 30(1):426–42. 10.1080/26410397.2022.2095707 PMC9542727 36169648

[38] Nyblade L Ndirangu JW Speizer IS Browne FA Bonner CP Minnis A Stigma in the health clinic and implications for PrEP access and use by adolescent girls and young women: conflicting perspectives in South Africa. BMC Public Health. (2022) 22(1):1–11. 10.1186/s12889-022-14236-z 36242000 PMC9563466

[39] Stoebenau K Muchanga G Ahmad SSO Bwalya C Mwale M Toussaint S Barriers and facilitators to uptake and persistence on prep among key populations in southern province, Zambia: a thematic analysis. BMC Public Health. (2024) 24(1):1–16. 10.1186/s12889-024-19152-y 38886691 PMC11184712

[40] For the provision of oral pre-exposure prophylaxis (prep) to persons at substantial risk of HIV infection 2 2021 Accessed Guidelines PrEP Final.

[41] Indlela Behavioural Hub (B-Hub)—Indlela [Internet]. Available online at: https://indlela.org/indlela-behavioural-insights-test-bit-consumer-panel/ (Accessed February 23, 2025)

[42] Acceptability of Intervention Measure (AIM), Intervention Appropriateness Measure (IAM), & Feasibility of Intervention Measure.

[43] Lewis JR. The system usability scale: past, present, and future. Int J Hum Comput Interact. (2018) 34(7):577–90. 10.1080/10447318.2018.1455307

[44] Brooke J. SUS: a “quick and dirty” usability scale. In: Jordan PW Thomas B Weerdmeester BA McClelland IL, editors. Usability Evaluation in Industry. London: Taylor & Francis (1996). p. 189–94. 10.1201/9781498710411-35

[45] Hyzy M Bond R Mulvenna M Bai L Dix A Leigh S System usability scale benchmarking for digital health apps: meta-analysis. JMIR Mhealth Uhealth. (2022) 10(8):e37290. 10.2196/37290 35980732 PMC9437782

[46] Cheah MH Gan YN Altice FL Wickersham JA Shrestha R Salleh NAM Testing the feasibility and acceptability of using an artificial intelligence chatbot to promote HIV testing and Pre-exposure prophylaxis in Malaysia: mixed methods study. JMIR Hum Factors. (2024) 11(1):e52055. 10.2196/52055 38277206 PMC10858413

[47] Ntinga X Musiello F Keter AK Barnabas R van Heerden A. The feasibility and acceptability of an mHealth conversational agent designed to support HIV self-testing in South Africa: cross-sectional study. J Med Internet Res. (2022) 24(12):e39816. 10.2196/39816 36508248 PMC9793294

[48] Phiri M Munoriyarwa A. Health chatbots in Africa: scoping review. J Med Internet Res. (2023) 25:e35573. p. 1–8. 10.2196/35573 35584083 PMC10337242

[49] Nadarzynski T Lunt A Knights N Bayley J Llewellyn C. “But can chatbots understand sex?” attitudes towards artificial intelligence chatbots amongst sexual and reproductive health professionals: an exploratory mixed-methods study. Int J STD AIDS. (2023) 34(11):809–16. 10.1177/09564624231180777 37269292 PMC10561522

[50] Alam L Mamun TI Mueller ST. Application of cognitive empathy elements into AI chatbots: an interview study exploring patient-physician interaction. J Cogn Eng Decis Mak. (2024) 0(0):1–19. 10.1177/15553434241310500

[51] Furini M Mariani M Montagna S Ferretti S. Conversational skills of LLM-based healthcare chatbot for personalized communications. In: Proceedings of the 2024 International Conference on Information Technology for Social Good (GoodIT ‘24); 2024 Sep 4–6; Bremen, Germany. New York (NY): Association for Computing Machinery (2024). p. 429–32. 10.1145/3677525.3678693

[52] Hodson N Williamson S. Can large language models replace therapists? Evaluating performance at simple cognitive behavioral therapy tasks. JMIR AI. (2024) 3:e52500. 10.2196/52500 39078696 PMC11322688

[53] Moore J Grabb D Agnew W Klyman K Chancellor S Ong DC Expressing stigma and inappropriate responses prevents LLMs from safely replacing mental health providers. The 2025 ACM Conference on Fairness, Accountability, and Transparency (FAccT ‘25); June 23–26, 2025; Athens, Greece (2025). p. 1. Available online at: http://arxiv.org/abs/2504.18412 (Accessed June 11, 2025)

[54] Ilicki J. A framework for critically assessing ChatGPT and other large language artificial intelligence model applications in health care. Mayo Clinic Proceedings: Digital Health. (2023) 1:185–8. 10.1016/j.mcpdig.2023.03.006 40206723 PMC11975638

[55] Nashwan AJ Abujaber AA Choudry H. Embracing the future of physician-patient communication: gPT-4 in gastroenterology. Gastroenterol Endosc. (2023) 1(3):132–5. 10.1016/j.gande.2023.07.004

[56] Du J Yuen C Slaughter M Chen AT. Perceived usability and experience with digital tools in the context of digital humanities research. Proc Assoc Inf Sci Technol. (2021) 58(1):435–9. 10.1002/pra2.474

[57] Kaur E Haghighi PD. A context-aware usability model for mobile health applications. ACM International Conference Proceeding Series [Internet] (2016). p. 181–9. Available online at:Available online at: /doi/pdf/10.1145/3007120.3007135?download=true (Accessed December 8, 2025)

[58] Bellamy RKE Dey K Hind M Hoffman SC Houde S Kannan K AI fairness 360: An extensible toolkit for detecting, understanding, and mitigating unwanted algorithmic bias. *ArXiv*. (2018) 63(4):1–15.

[59] Zarei N Joulaei H Darabi E Fararouei M. Stigmatized attitude of healthcare providers: a barrier for delivering health services to HIV positive patients. Int J Community Based Nurs Midwifery. (2015) 3(4):292–300. 26448956 PMC4591575

[60] Young SD Hlavka Z Modiba P Gray G Van Rooyen H Richter L HIV-Related Stigma, social norms and HIV testing in soweto and vulindlela, South Africa: nIMH project accept (HPTN 043) NIH public access. J Acquir Immune Defic Syndr. (2010) 55(5):620–4. 10.1097/QAI.0b013e3181fc6429 20980913 PMC3136617

[61] Yu H. Social stigma as a barrier to HIV testing: evidence from a randomized experiment in Mozambique. J Dev Econ. (2023) 161:103035. 10.1016/j.jdeveco.2022.103035

[62] Izadi S Forouzanfar M. Error correction and adaptation in conversational AI: a review of techniques and applications in chatbots. AI. (2024) 5(2):803–41. 10.3390/ai5020041

[63] Xue J Wang YC Wei C Liu X Woo J Kuo CCJ. Bias and Fairness in Chatbots: An Overview. (2023). Available online at: http://arxiv.org/abs/2309.08836 (Accessed October 11, 2024)

[64] Mao W Qiu X Abbasi A. LLMs and their applications in medical artificial intelligence. ACM Trans Manag Inf Syst. (2025) 16(2):7. 10.1145/3711837

[65] View of Human in the loop requirement and AI healthcare applications in low-resource settings: A narrative review. Available online at: https://samajournals.co.za/index.php/sajbl/article/view/1975/1074 (Accessed June 11, 2025)

