# Supplementary material for: Breaking barriers: harnessing artificial intelligence for a stigma-free, efficient HIV prevention assessment among adults in South Africa
Source: Front Digit Health. 2026 Feb 2;7:1731002. doi: 10.3389/fdgth.2025.1731002 (PMC12908171; doi:10.3389/fdgth.2025.1731002)
Supplement: Supplementary file 1 [file Datasheet1.pdf]

Design, Technical Development, and Iterative Refinement of the Your Choice Conversational Agent

The Your Choice conversational agent, delivered via an Android app, was designed using a production-oriented LLM engineering approach informed by structured user feedback and researcher review. Each iteration combined engineering activities, including prompt engineering, the introduction and refinement of system-level guardrails, the development of monitoring instrumentation, and feature enhancements, with changes validated through internal Audere testing before redeployment. Feedback from recipients of care and healthcare providers was translated into concrete engineering actions, such as prompt restructuring, persona tuning, performance optimizations, and new input/output modalities, ensuring that improvements were measurable, auditable, and safe. This continuous loop allowed the engineering team to, over time, improve accuracy, contextual viability, empathy, and usability while minimizing regression risk as model upgrades, prompts, and features evolved.

Below is a description of technical development activities:

Baseline prompt architecture and persona design

- Implemented a structured system prompt defining:
- A South Africa based, empathetic clinical persona
- Definition of allowed and disallowed topics (e.g. HIV risk, PrEP; exclusion of PII and unrelated topics) to manage conversation flow and guide users towards HIV prevention outcomes
- Prompt for summarizing conversations to guide LLMs to extract relevant patient details, needs, and clinically relevant information in concise paragraph
- Designed prompts to be portable across multiple LLMs to enable model comparison and future flexibility

Instrumentation and observability

- Developed logging at the message and conversation level, including:
  - Full conversation transcripts for ROC/LLM interactions
  - LLM-generated summaries for HCP
  - LLM token usage for cost measurement and latency metrics
  - Conversation length and drop-off points
  - Enabled post-hoc analysis of prompt changes on cost, performance, and response quality.

After each testing cycle, based on issues identified:

- Reviewed transcripts flagged for misinformation, bias, abrupt tone, or poor conversation flow
- Where needed, adjusted prompt wording, ordering, and context provided (e.g. number of last messages provided to LLM for context)
- Tuned conversation steering logic to balance efficiency with empathetic pacing
- Prompt updates were versioned and compared against prior versions to detect regressions.

Accuracy and viability validation

- Developed a first-generation evaluation rubric to score conversations and summaries across:
  - Clinical correctness
  - Risk of misinformation or harm
  - Contextual appropriateness for South Africa
- Used labelled “good” and “bad” conversations to test prompt changes and ensure updates stayed within defined quality thresholds.

Cross-model normalization

- Tested the same prompt structures across multiple LLMs to assess:
    - Sensitivity of outputs to prompt wording
    - Differences in hallucination rates and tone
- Identified model that was unsuitable for live testing due to persistent hallucinations (Meta Llama)
- Built an initial automated test harness to re-run validation datasets whenever:
    - Prompts were modified
    - Models were upgraded
- Implemented voice-to-text and text-to-audio capabilities leveraging on device Android code libraries to reduce latency and cost in cloud processing of audio, and due to varying availability of multi-modal input processing support

Performance and cost optimization investments

- Optimized prompt length and response timing
- Logged cost per conversation by model and prompt version
